# Unleashing Natural Killer Cells in the Tumor Microenvironment–The Next Generation of Immunotherapy?

**DOI:** 10.3389/fimmu.2020.00275

**Published:** 2020-02-21

**Authors:** Aviad Ben-Shmuel, Guy Biber, Mira Barda-Saad

**Affiliations:** Laboratory of Molecular and Applied Immunology, The Mina and Everard Goodman Faculty of Life Sciences, Bar-Ilan University, Ramat Gan, Israel

**Keywords:** natural killer cells, inhibitory checkpoints, immunotherapy, tumor microenvironment, tumor ligands

## Abstract

The emergence of immunotherapy for cancer treatment bears considerable clinical promise. Nevertheless, many patients remain unresponsive, acquire resistance, or suffer dose-limiting toxicities. Immune-editing of tumors assists their escape from the immune system, and the tumor microenvironment (TME) induces immune suppression through multiple mechanisms. Immunotherapy aims to bolster the activity of immune cells against cancer by targeting these suppressive immunomodulatory processes. Natural Killer (NK) cells are a heterogeneous subset of immune cells, which express a diverse array of activating and inhibitory germline-encoded receptors, and are thus capable of directly targeting and killing cancer cells without the need for MHC specificity. Furthermore, they play a critical role in triggering the adaptive immune response. Enhancing the function of NK cells in the context of cancer is therefore a promising avenue for immunotherapy. Different NK-based therapies have been evaluated in clinical trials, and some have demonstrated clinical benefits, especially in the context of hematological malignancies. Solid tumors remain much more difficult to treat, and the time point and means of intervention of current NK-based treatments still require optimization to achieve long term effects. Here, we review recently described mechanisms of cancer evasion from NK cell immune surveillance, and the therapeutic approaches that aim to potentiate NK function. Specific focus is placed on the use of specialized monoclonal antibodies against moieties on the cancer cell, or on both the tumor and the NK cell. In addition, we highlight newly identified mechanisms that inhibit NK cell activity in the TME, and describe how biochemical modifications of the TME can synergize with current treatments and increase susceptibility to NK cell activity.

## Introduction

### NK Cells and Cancer

In recent years, the field of immunotherapy has emerged as one of the most promising approaches for treating cancer (1). Though most immunotherapies have traditionally focused on T-cells, NK cell-based therapies are rapidly emerging in research and in the clinic (2, 3).

NK cells are cytotoxic innate lymphoid cells (ILCs), which can target and eliminate cancer cells through secretion of cytolytic granules, and trigger an immune response via secretion of immunomodulatory cytokines (4). In contrast to T- and B-cells, NK cells express a multitude of intrinsic germline-encoded activating and inhibiting membrane receptors, and therefore do not require antigen specificity (5, 6). Immune-editing of the tumor promotes evasion from the anti-tumor immune response; a common remodeling event is downregulation of β2-microglobulin, which leads to reduced MHC presentation. The selective pressure leading to low MHC presentation impairs T-cell anti-tumor activity (7). NK cell function is therefore partially complementary to T-cells, as they can target and lyse MHC-I deficient cells, in a process known as “missing-self recognition” (8, 9). In addition to missing-self recognition, activation of NK cells relies on the equilibrium between activating and inhibitory signals derived from surface receptors engaged with cognate ligands on target cells (10, 11). Central activating and co-activating NK cell receptors include the natural cytotoxicity receptors (NCRs) NKp46, NKp30, and NKp44, CD16, NKG2D, NKG2C, DNAX Accessory Molecule-1 (DNAM-1), and 2B4 (12, 13). In parallel, the important inhibitory receptors on NK cells engage with MHC-I ligands to down modulate the NK cell response, and these include the Killer-cell immunoglobulin-like receptors (KIRs), and the CD94/NKG2A heterodimer (12). NK cells express additional checkpoint inhibitory receptors, which play important roles in constraining their activity when engaged with cognate ligands, as will be discussed below.

Various approaches have been developed to bolster NK cell activity against cancer, some of which are being utilized in pre-clinical and clinical trials (3, 14). Significant hurdles still persist, however, for immunotherapeutic treatments in general, and for NK cells in particular. These include concerns regarding potential autoimmune cytotoxicity for therapies such as cytokine administration and immune checkpoint inhibitors, the magnitude of the patient response to treatment, and patient relapse due to innate or acquired resistance (15–18). Moreover, though some NK cell based treatments have shown promising results for hematological malignancies, NK cells generally have low capacity to infiltrate solid tumors, and so far, the efficacy against advanced cancers and solid tumors remains relatively low (19). Cytolytic immune cells such as NK cells and CD8^+^ T-cells are also suppressed through multiple pathways in the tumor microenvironment (TME) (20, 21).

Recent comprehensive reviews provide detailed descriptions and delineate the obstacles remaining in multiple NK-based treatments that have been developed and tested in pre-clinical and clinical trials. These reviews cover topics including cytokine therapy, hematopoietic stem cell transplantation (HSCT), adoptive cell transfer, and CAR-NK therapy (2, 3, 22–25). Here, we summarize recent pre-clinical and clinical evidence regarding NK cell expression of checkpoint molecules and some of the most recent NK-based immunotherapeutic strategies. These approaches include targeting NK surface receptors, NK cell ligands on tumors, and identification and modulation of pathways in the TME for sensitization to NK cell activity. We highlight newly developed technologies that may increase NK cell activation, infiltration, survival, and proliferation.

### Triggering of Adaptive Immunity and Cancer Immune Surveillance by Subsets of NK Cells

Besides their potential for direct elimination of cancer cells, the crosstalk between NK cells and additional immune cells such as T-cells and dendritic cells (DCs) in the TME initiates potent anti-tumor effects. This interplay between NK cells and DCs can promote DC uptake of tumor antigens in secondary lymph nodes for presentation to and activation of T-cells, which facilitate additional anti-tumor responses (23). Recently, it was shown that NK cells recruit conventional type 1 DCs through secretion of the chemo attractants CCL5, XCL1, and XCL2, and the extent of this process correlates with cancer patient survival (26). Furthermore, production of the cytokine FLT3L by NK cells increased the frequency of conventional type 1 DCs in tumors, and frequencies of NK cells and conventional type 1 DCs correlate with responses to anti-PD-1 therapy (27). Moreover, in a recently established murine model of lung adenocarcinoma, expression of NK cell ligands on tumors and their activation facilitated the adaptive response of tumor-specific T-cells and reduction in tumor growth (28). These studies and others (29, 30) illustrate some of the indirect roles NK cells may play in mediating the immune response in the TME, warranting development of novel NK-based immunotherapies.

NK cells are not a uniform immune cell subset, but rather very heterogeneous, and can be segregated into subsets with distinct tissue residency, phenotypes, and functional activity. NK cells are traditionally classified into the CD56^bright^ CD16^dim^ subset and CD56^dim^ CD16^bright^ subset. CD56^bright^ CD16^dim^ cells are generally found in certain peripheral tissues (such as lymph nodes, gut, tonsils, uterus, and skin) and considered immature, with low KIR expression and high expression of CD94/NKG2A; these cells primarily secrete chemokines and cytokines such as IFNγ and TNFα (13, 31, 32). The CD56^dim^ CD16^bright^ subset is the major subset in peripheral blood and is considered terminally differentiated with high KIR expression; these cells exert their cytotoxic activity through secretion of perforin and granzyme (33). Over the last few years, additional NK cell subpopulations with distinct phenotypic expression profiles have been further identified and classified in different tissues (34) and in both solid and hematological malignancies (35). In addition to these findings, the characterization of ILCs over the past decade has demonstrated overlapping surface marker expression between NK cells and other ILC subsets in certain tissues (such as ILC1 and 3) (36). ILC1s, which have poor cytotoxic capacity but potent cytokine secretion share multiple common markers with NK cells, including NKp46 (37). Therefore, due to the similarity between ILCs and NK cell subtypes, their classification has considerable importance in the prism of immunotherapy for the specificity of treatment. Recently, it was shown that NK cells can be converted into ILC1s in the TME by a TGF-β dependent mechanism, and these converted ILC1s lose the capacity to control tumor growth and dissemination, and may promote tumorigenicity (38). The plasticity of NK cells in response to TGF-β is further exemplified by evidence of their conversion to decidual NK-like cells in the TME, which promote angiogenesis (39). The heterogeneity and plasticity of NK subsets adds an additional layer of complexity to treatment, yet it also opens doors toward fine tuning and enhancing the NK-based response; in the case of TGF-β mediated conversion, targeting TGF-β pathways (as discussed below) or identifying the intracellular pathways leading to NK cell conversion can offer new therapeutic opportunities. Further identification of NK cell/ILC markers and phenotypes, as well as molecular mechanisms regulating their effector function during tissue homeostasis or disease should greatly contribute to future therapeutic efficacy.

## The Tumor Microenvironment Restrains NK Cell Activity

The extent of NK cell infiltration into solid tumors is an intensively debated subject, though there is consensus that such infiltration may contribute to improved clinical prognosis (40–42). Though some tumors, such as colorectal cancers, may almost completely exclude NK cell subsets (43), others such as breast, renal, lung, and head and neck squamous cell carcinoma (HNSCC) tumors are infiltrated by NK cells with possible clinical benefit (44–47). Poor NK cell homing and infiltration to the TME may be attributed to interference with NK chemotactic signaling and activation, and physical properties of the tumor bed such as vasculature density and ECM composition (40, 48–50).

Inside the tumor, immune cells are confronted with a suppressive milieu. The TME harbors an assortment of cell types that down modulate the immune response, including stromal cells, fibroblasts, regulatory T-cells (Tregs), and myeloid derived suppressor cells (MDSCs) (51, 52) which can induce specific changes in cytotoxic lymphocyte phenotype, metabolic program, transcriptional profile, and epigenetic profile (21, 53, 54). The TME induces inhibitory effects on immune cells through a variety of processes, specifically chronic immune cell activation and an inflammatory microenvironment, secretion of immunomodulatory cytokines and soluble factors, induction of hypoxia, and upregulation of inhibitory checkpoint ligands, which down-regulate immune cell activation when engaged with immune checkpoint receptors, such as Programmed cell death protein 1 (PD-1) and cytotoxic T-lymphocyte-associated protein 4 (CTLA-4) (51, 55). For example, MDSCs (which include myeloid progenitor cells and immature mononuclear cells) are recruited to the TME and can induce immune cell down-modulation through production of arginase, nitric oxide, TGF-β, and IL-10 (56). Tumor-derived macrophages are also recruited to the TME, and can suppress immune cells via secretion of TGF-β and IL-10. They can also recruit Tregs, which have immune-suppressive properties (57, 58). Tregs-enriched in the TME can inhibit CD8^+^ T-cells and NK cells directly or via secretion of TGF-β, and IL-10 (59, 60), inducing upregulation of inhibitory checkpoint receptors (61). Although IL-10 has immunosuppressive effects on NK cells, it has pleotropic effects on the regulation of tumor-promoting inflammation, and thus has been tested in clinical trials to augment CD8^+^ T-cell function against cancer (62). Therefore, it appears that multiple immunosuppressive pathways in the TME collaborate in the down-modulation of immune cell activity.

Exhausted T-cells demonstrate impaired effector functions including reduced cytotoxicity and cytokine secretion, impaired proliferation, reduced responses to cytokine stimulation, upregulation of inhibitory checkpoint receptors, and may ultimately undergo apoptosis (63–68). NK cells also undergo functional exhaustion when exposed to the conditions prevalent in the TME. These phenotypes in NK cells are induced by high expression of checkpoint ligands on tumors (such as Programmed death-ligand 1 (PD-L1) and HLA-E) (69, 70), inhibitory cytokines and inhibitory soluble factors (such as TGF-β and IL-10) (71–73), hypoxia (74, 75), exposure to tumor suppressor cells (i.e., Tregs, tumor associated macrophages, and MDSCs) (76–78), and sustained chronic activation (such as sustained activation through the activating NKG2D receptor) (79, 80).

### Checkpoint Receptors Modulating NK Cell Activity

Exhausted NK cells share some phenotypes with exhausted CD8^+^ T-cells, namely downregulation of effector cytokines such as IFN-γ, impaired degranulation and cytotoxicity, downregulation of activating receptors such as NKG2D, upregulation of inhibitory receptors such as NKG2A, and transcriptional changes that promote exhaustion, i.e., downregulation of transcription factors including Eomesodermin and T-bet (20, 81–86). Recent studies further characterized inhibitory checkpoint receptors that promote NK cell dysfunction during malignancy. These studies show that NK cells, in addition to T-cells, can contribute to the efficacy of inhibitory checkpoint inhibition (ICI) through direct or indirect anti-tumor immunity. Therefore, understanding which checkpoint receptor is upregulated on specific NK subsets, and how such upregulation modulates the cell's capacity to generate an immune response, provides valuable information for treatment and combinatorial strategies. Table 1 lists some of the markers found on NK cells that may act as inhibitory checkpoint molecules.

**Table 1:** NK cell inhibitory checkpoint receptors: Expression on NK cells and modulation of NK cell effector functions in different cancers-evidence from patients, animal models, and *in-vitro* studies.

**NK cell Marker**	**Experimental Systems-NK cell markers from patients/animal models/*in-vitro* induction**
PD-1	**Patients** Head and Neck cancer patients (69) Anaplastic thyroid cancer patients (87) Hodgkin lymphoma/diffuse large B-cell lymphoma patients (88) Gastric cancer patients (89) Kaposi sarcoma patients (90) Renal cell carcinoma patients (91) Multiple Myeloma patients (92) ***In-vitro*** **studies** Breast cancer cell lines (93)
TIM-3	**Patients** Metastatic melanoma patients (94–96) Lung adenocarcinoma patients (97) Colorectal cancer patients (96, 98) Bladder cancer patients (96, 99) Endometrial cancer patients (100) Esophageal cancer patients (101) **Animal models** Murine lung metastases model (96) Murine esophageal carcinoma model (101)
TIGIT	**Patients** Colon cancer patients (102, 103) Myelodysplastic Syndrome patients (104) **Animal models** Colon/breast/melanoma murine models (103) ***In-vitro*** **studies** Fap2 mediated inhibiton (102) Monocyte and MDSC co-culture (104) Breast cancer cell lines (105)
CD96	**Patients** Hepatocellular carcinoma patients (106) **Animal models** Murine melanoma and fibrosarcoma models (107) Murine melanoma, lung carcinoma, prostate carcinoma, colon carcinoma, and breast tumor models (108, 109)
NKG2A	**Patients** Breast cancer patients (110) Neuroblastoma patients (111) CLL patients (high HLA-E expression) (112) Head and neck, Squamous cell carcinoma, colorectal carcinoma (46) **Animal models** B/T-cell lymphoma murine models (46) ***In-vitro*** **studies** Upregulation following cytokine induction (NKs from multiple myeloma patients) (113) Erythroleukemia, B-cell lymphoma, head and neck, squamous cell carcinoma, ovarian tumor cell lines (46)

#### PD-1

PD-1 is an inhibitory checkpoint molecule expressed by activated T-cells (114, 115), and was also shown to be expressed on NK cells (116, 117). It marks CD56^dim^NKG2A^−^KIR^+^CD57^+^ mature NK cells from Human Cytomegalovirus (HCMV) seropositive subjects (117), and may indicate an exhausted NK cell subset with memory-like features (118). PD-1 expression on NK cells is upregulated in several cancers, including head and neck cancer (69), thyroid cancer (87), Hodgkin lymphoma (HL) (88), digestive cancers (esophageal, liver, colorectal, gastric and biliary) (89), breast cancer (93), NK/T cell lymphomas (119), Kaposi sarcoma (90), renal cell carcinoma (91), and multiple myeloma (92). Such upregulated expression of PD-1 by NK cells in the TME is associated with the down-modulation of NK cell activity, manifested by reductions in cytotoxicity, cytokine secretion (e.g., IFN-γ, TNF-α, and GM-CSF), and proliferation (20). PD-1 blockade can unleash T-cells against PD-L1-expressing tumors; however, MHC-I loss on the tumor surface can impact the efficacy of treatment. Therefore, contribution of NK cells also appears important in PD-1 blockade, especially in the context of MHC-I loss on tumors. Indeed, PD-1/PD-L1 blockade in mice bearing PD-L1^+^ MHC-I^−^ tumors demonstrated the importance of NK cells for the efficacy of these treatments (120). Interestingly, some PD-L1 negative tumors respond to anti-PD-L1 therapy, and a recent study demonstrated that this effect may be mediated by PD-L1^+^ NK cells. PD-L1^+^ NK cells treated with anti-PD-L1 showed enhanced activation and effector function, possibly identifying a novel biomarker of the NK PD-L1^+^ subset for immunotherapy (121).

#### TIM-3

Activation of T-cell immunoglobulin and mucin-domain containing-3 (TIM-3) by antibody cross-linking initially showed significant decrease of NK cell function (122), and its expression marks mature and exhausted NK cells (122). TIM-3^+^ NK cells isolated from peripheral blood of metastatic melanoma patients are functionally exhausted, and inhibitory antibodies against TIM-3 can reverse this NK cell dysfunction (94, 95). Higher expression of TIM-3^+^ NK cells is also apparent in lung adenocarcinoma with lymph node metastases at the progressive tumor stage, and is correlated with decreased patient survival (97). Here, as well, blocking TIM-3 with antibodies increased NK cell cytotoxicity and cytokine secretion. Additional recent studies identified TIM-3 expression as a marker of NK cell dysfunction and disease severity in colorectal cancer, esophageal cancer, endometrial cancer, and bladder cancer (96, 98–101). Interestingly, TIM-3 engagement was initially shown to increase the expression of IFN-γ by NK cells in response to galectin-9, the β-galactoside binding lectin (123). Since TIM-3 can bind additional ligands [such as phosphatidylserine and CEA-related cell adhesion molecule-1 (124, 125)], its activity, whether stimulatory or repressive, may be ligand dependent. Therefore, additional characterization of TIM-3 ligands and their downstream signaling mechanisms is needed for a better understanding of its targeting in immunotherapy. TIM-3 upregulation may also be associated with adaptive resistance to PD-1 blockade, emphasizing its importance as an alternative checkpoint, which should be considered in patients with anti-PD-1 resistant tumors (126). Therefore, anti-TIM-3 monoclonal antibodies such as TSR-022 (NCT02817633, NCT03680508) and Sym023 (NCT03311412) are under evaluation in clinical trials in combination with anti-PD-1 therapy for advanced solid tumors.

#### TIGIT

T cell immunoreceptor with Ig and ITIM domains (TIGIT) is a suppressive receptor in T-cells (127) that was recently shown to contribute to NK cell dysfunction in cancer. TIGIT is expressed on activated human NK cells, competing for the DNAM-1 ligands PVR/CD155 and PVRL2/CD112 (128) and thereby downregulating NK cell activation. Recent studies demonstrated that blockade of TIGIT significantly enhances anti-tumor NK cell activity against breast cancer and endometrial cancer (100, 105), and can bypass MDSC-mediated supression (104). Interestingly, NK cell inhibition through the TIGIT receptor in the tumor microenvironment may also contribute to carcinogenesis by binding to inhibitory ligands of the onco-bacteria, *Fusobacterium nucleatum* (FN) (102). The Fap2 protein expressed on FN directly binds TIGIT on NK cells in colon adenocarcinoma tumors, reducing NK cell activity and enabling tumor evasion. Though TIGIT clearly suppresses NK cell activity, the role it plays in maintaining NK cell tolerance in cancer was not fully understood until recently. Zhang et al. revealed that TIGIT expressing NK cells are a functionally exhausted subset in colon cancer and mouse models of melanoma and breast cancer (103). TIGIT^+^ NK cells demonstrated low cytotoxicity and cytokine production, a phenotype that was reversed in TIGIT-deficient mice or under TIGIT antibody blockade. Interestingly, blockade of TIGIT in NK cells alone appeared necessary for the most potent anti-tumor responses mediated by CD8^+^ T-cells, and generated a memory response against secondary tumor challenge. This requires further study, but raises the exciting possibility of memory responses against cancer mediated by NK cell activity. Since TIGIT blockade synergizes with anti-PD-1 treatment (129), it is also undergoing testing in combination in multiple clinical trials for solid tumors (NCT04047862, NCT03563716, NCT03628677).

#### CD96

T cell activation, increased late expression (tactile)/CD96 shares sequence similarity to TIGIT, and promotes NK cell adhesion by binding CD155 (130). More recently, it was also shown to down modulate NK cell activity (107). CD96-deficient mice are resistant to carcinogenesis and lung metastases (107), and blocking CD96 unleashes NK cell activity in mouse models of lung metastases, significantly suppressing metastatic growth (108, 109). Recent work by Sun et al. identified a subset of CD96^+^ NK cell infiltrates in hepatocellular carcinoma associated with a transient increase in disease-free survival in patients, and enhanced overall survival (106). This NK cell subset had low production of IFNγ and TNFα, as well as low perforin and granzyme B gene expression. Mechanistically, TGF-β1 appears to sustain CD96 expression on NK cells from hepatocellular carcinoma patients, further emphasizing its role in promoting NK cell exhaustion in the TME. These data potentially suggest a dual CD96/TGF-β1 targeting approach (106) which may also synergize with anti-TIGIT blockade, as they both share the CD155/CD112 ligands, and both may provide a synergistic effect with other immune checkpoint receptors such has PD-1 (103). It is interesting to speculate that CD96^+^, like TIGIT^+^ NK cells represent an exhausted subset, and the interplay between TIGIT/CD96 and DNAM-1 expression may provide a credible biomarker for identifying and characterizing exhausted NK cells in the TME.

#### LAG-3

The inhibitory function of Lymphocyte-activation gene 3 (LAG-3) on NK cells is less well-characterized. LAG-3 is expressed on activated NK cells, binding MHC II, and the C-type lectin receptor, LSECtin, which is upregulated on various types of tumors (131–133). Early reports based on LAG-3 deficient mice showed that NK cells from these mice were defective in their ability to lyse certain tumor targets, suggesting a role for this receptor in facilitating NK cell killing (134). However, MHC-I mismatched targets were successfully lysed by NK cells from these LAG-3 deficient mice. Moreover, blocking LAG-3 with mAbs had no effect on NK cell lysis of various target cells (135). This may suggest a role for LAG-3 in the NK cell maturation and receptor repertoire, affecting the cell's capacity to distinguish between MHC-I matched healthy and transformed cells, but not in directly inhibiting NK cell killing efficacy. By contrast, a recent report showed that chronic stimulation of adaptive NK cells from HCMV seropositive donors via NKG2C and IL-15, NKp30, or NKG2D increased PD-1 and LAG-3 surface expression, downregulating NK activity during subsequent interactions with tumor targets (136). These experiments provide primary evidence of LAG-3 as a potential exhaustion marker on NK cells following chronic stimulation. It would be interesting to speculate that these NK cells could have their cytotoxic capacity restored by blocking LAG-3 on their surface. This would provide further insight regarding its function on the NK cell surface. Accordingly, blocking LAG-3 in a murine lung metastases model restored the capacity of NK cells to clear metastasis (137). In line with these findings, a role for LAG-3 was also recently demonstrated in NK cells with a deficiency in Wiskott-Aldrich syndrome protein (WASp). WASp is a critical actin nucleation promoting factor in hematopoietic cells, and along with the WASp interacting protein (WIP), plays a critical role in NK cell activation (138–142). WASp-deficient NK cells display deficient effector functions against tumor targets, and were shown to express increased exhaustion markers, including LAG-3 (143). Thus, it is appears that LAG-3 expression correlates with low overall NK cell activation status and effector function, and NK cells could contribute to the effects of anti-LAG-3 mAb therapy. Several anti-LAG-3 mAbs are being tested in multiple clinical trials for advanced solid tumors, either alone, or in combination with anti-PD-1 (3) (e.g., NCT03489369, NCT04080804, NCT03250832, NCT01968109).

#### KIR/NKG2A

HLA-E, a ligand for CD94/NKG2A is upregulated in certain cancers (144), and tumors can inhibit NK cells through expression of HLA molecules (145). Targeting the KIR and CD94/NKG2A inhibitory NK cell receptors to generate missing-self recognition is therefore another primary strategy to unleash NK cell activity (84, 110–113, 146, 147). Drugs introduced to block these inhibitory molecules, such as antibodies against KIRs- Lirilumab, which targets KIR2DL1-3 and KIR2DS1/2, and IPH4102, which targets KIR3DL2, are currently being tested in various clinical trials (https://clinicaltrials.gov) (146, 148, 149). In pre-clinical models, anti-KIR antibodies showed potent effects on NK cell activity (145, 150, 151). Lirilumab is effective in AML and multiple myeloma (MM) patients with low toxicity and minimal adverse events (152, 153). In the study by Vey et al., however, Lirilumab treatment did not significantly improve leukemia-free survival in AML (154), and no clinical efficacy was observed in a phase II trial of smoldering MM (155). Interestingly, Carlsten et al. suggest that one possible mechanism impeding the efficacy of this treatment is the downregulation of KIR2D on the NK cell surface following infusion of anti-KIR antibodies. Since NK cell licensing requires engagement of KIR receptors with cognate MHC-I ligands (156, 157), administration of anti-KIR mAbs may counterintuitively lead to down-modulation of NK cell activity (158). One possible solution involves lower or alternating dosing regimens (30). Furthermore, anti-KIR mAbs can synergize well with other mAb therapies such as Rituximab and anti-PD-1 (151, 159). Additional evaluation of Lirilumab for clinical use is warranted, and it is currently being tested as a combination treatment for various malignancies, including bladder cancer (NCT03532451) and advanced and metastatic tumors (NCT03203876, NCT01714739, NCT03347123).

The inhibitory NKG2A receptor was also shown to be a beneficial target for NK-based therapy. NKG2A targeting may be especially significant given the higher HLA-E expression on certain cancerous tissues relative to other classical HLAs (85, 144, 160). Recent work from the Vivier group has shown the potent effect of anti-NKG2A antibody (Monalizumab) on NK cell and CD8^+^ cell effector functions. Monalizumab increases NK and CD8^+^ antibody-dependent cellular cytotoxicity (ADCC) and synergizes with anti-PD-1/PD-L1 blockade and anti-epidermal growth factor receptor (EGFR) (Cetuximab) treatment (46). Interim results from clinical studies of Monalizumab + Cetuximab/ Durvalumab (anti-PD-L1) appear well-tolerated, with encouraging efficacy profiles (NCT02643550, NCT02671435) (46, 161).

## Targeting Tumors for Reinvigoration of NK Cell Activity

Toxicities and poor patient response to immunotherapies, especially against advanced/solid tumors, remain challenging (15, 162). Non-specific augmentation of immune cell activity can generate immune-related adverse events, and advanced tumors have powerful immunosuppressive properties and are difficult to infiltrate (163, 164). For example, cytokine therapy, such as administration of IL-2, 12, 15, and 21 can promote the proliferation, survival, and activation of NK cells *in-vitro* and *in-vivo*. However, their use in clinical trials has shown dose-dependent toxicities such as hypotension, thrombocytopenia, neutropenia, and an increase of Alanine transaminase/Aspartate transaminase (ALT/AST) levels. NK cells, in addition to other immune cell subsets such as T-cells, may also be involved in some of these toxic side effects (165–168). In addition, though therapies targeting inhibitory checkpoint receptors expressed on NK cells, such as anti-PD-1 and the PD-1 ligand, PD-L1, increase NK cell activity (121, 169, 170), they may inadvertently cause various dose-dependent immune-related adverse events, such as pneumonitis, colitis, hepatotoxicity, endocrinopathies, and neurological and cardiac toxicities (171).

**Figure 1:**
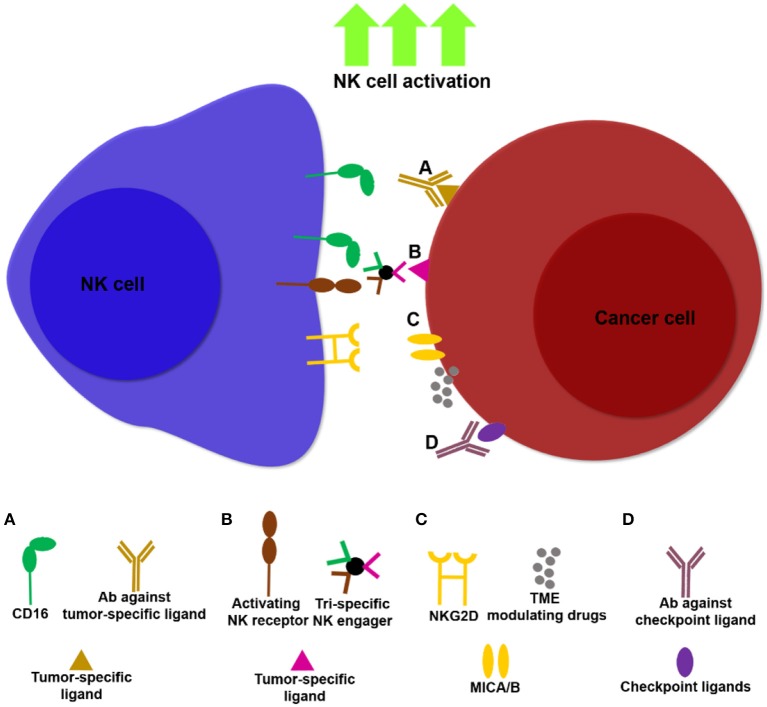
Examples of NK cell immunotherapies targeting NK cells and tumors. Multiple approaches are being developed to unleash NK cell activity against tumors, thereby increasing potency and specificity of NK cell based treatments. **(A)** Antibodies that bind tumor ligands (e.g., anti-EGFR, anti-CD20) induce NK cell ADCC through ligation to CD16. **(B)** NK cell engagers can target multiple activating NK cell receptors in addition to CD16 (172), together with tumor ligands such as CD20, thereby facilitating NK cell activation and ADCC. **(C)** Drugs that modulate the TME, such as histone deactetylase inhibitors, induce upregulation of the NKG2D ligands, MICA/B, promoting NK cell infiltration and cancer cell lysis. **(D)** Masking tumor checkpoint ligands, such as PD-L1 and PCNA can unleash NK cell activity in the TME.

Harnessing modalities that can target ligands expressed predominantly on tumors and not on healthy tissues, rather than unleashing immune cell activity in a systemic fashion (e.g., systemic administration of cytokines and checkpoint inhibitors), have the potential to overcome some of these obstacles (Figure 1). Such strategies may increase the activation and infiltration of NK cell subsets *in-situ* by promoting localized anti-tumor immune responses.

### Antibodies to Direct NK Cell Activity Toward Cancer Cells

#### Antibodies for NK Cell Mediated ADCC

Monoclonal antibodies (mAbs) which target tumor-specific ligands have been used for treatment of hematological and solid malignancies for the last two decades (173). mAbs can have several mechanisms of action, including initiation of ADCC, mediated by engagement of the CD16 (FcγRIII) activating receptor on the NK cell with the Fc portion of the antibody that is directed toward the tumor ligand (174). The relative contribution of NK cells to the success of mAb therapies is not completely clear, but evidence of their role has increased in recent years (175). Several recent studies demonstrated enhanced efficacy of NK cell mediated ADCC using novel tumor-specific antibodies (Table 2).

**Table 2:** Antibody-based treatments reported to augment NK cell activity.

**Treatment**	**Key elements**	**Current stage of development**	
		**Pre-clinical**	**Clinical trials**
293C3-SDIE	Optimized anti-CD133 (high expression on colorectal cancer) antibody containing S239D and I332E amino acid substitutions, increasing affinity for CD16.	✓ (176)	
VAY736	Optimized anti-BAFF-R (highly expressed on B-ALL) antibody.		NCT03400176
CSL362/ Talacotuzumab	Humanized anti-CD123 monoclonal antibody (high expression on Hodgkin lymphoma) with increased affinity for CD16.		NCT03011034
B12	Anti-IL-7 receptor antibody (IL-7 promotes leukemia development and chemotherapy resistance). Demonstrates rapid internalization and lysosome trafficking.	✓ (177)	
MEN1112	Anti-CD157 antibody (high expression on primary AML cells).		NCT02353143
7C6 mAb	Monoclonal antibody directed against MICA α3 domain, preventing proteolytic shedding of MICA/MICB from tumors.	✓ (178)	
Elotuzumab	Antibody against SLAMF7 (high expression on multiple myeloma).		28 active/recruiting clinical trials as of February 2020 (www.clinicaltrials.gov)
F1	Antibody against the aspartic protease cathepsin D (high production and secretion by breast cancer cells).	✓ (179)	
hu14.18K322A	Anti-GD2 antibody (high expression on neuroblasts).		NCT02159443, NCT01857934
Codrituzumab	Antibody targeting oncofetal protein glypican-3 (high expression on hepatocellular carcinoma).		NCT01507168 (completed phase II)
BI 836858	Anti-CD33 antibody (high expression on AML cells).		NCT02240706, NCT02632721

##### Clinically utilized mAbs- recent evidence of NK cell contribution and optimization of treatment

*Anti-EGFR: cetuximab*. Cetuximab, is an anti-EGFR mAb used clinically for cancer treatment. Cetuximab can activate NK cell ADCC *in-vitro* and *in-vivo*, and initiate an adaptive immune response through activation of NK cell:DC crosstalk (180–182). *Ex-vivo* stimulation of NK cell ADCC with Cetuximab may also be predictive of clinical responses in patients (183). The efficacy of mAb treatment, including that of Cetuximab, is influenced by FCγR polymorphism (184). The homozygous genotype of valine at position 158 on the FcγRIIIa receptor (158 V/V genotypes), as opposed to the 158 V/F or 158 F/F FcγRIIIa genotypes is often, but not always, associated with improved clinical outcomes (185–189). Moreover, FCγ receptor affinity is impacted by patterns of antibody glycosylation (190) and by particular IgG variants. For example, though IgG1 and IgG3 appear to bind all Fcgamma receptors, IgG2 and IgG4 demonstrate varying affinity for specific Fcgamma receptors and their variants (191). In order to overcome these obstacles, much effort has been placed in IgG engineering to enhance the efficacy of ADCC. Glyco-engineering of antibodies, such as removal of fucose from the Fc region to generate non-fucosylated antibodies can significantly enhance interaction between antibodies and FcγRIIIa (192). Furthermore, screening for amino acid point mutations on antibody Fc regions which enhance ADCC has yielded mAbs which generate superior ADCC relative to their WT counterparts (190). Cytokine administration together with Cetuximab increases efficacy of NK cell activation and function (180, 193), and therefore Cetuximab was recently utilized in clinical trials for unresectable primary or recurrent HNSCC in conjunction with IL-12 administration (194). Combination treatment was generally well-tolerated. About half of the patients in this study demonstrated an average prolonged progression-free survival of 6.5 months, although neither complete nor partial responses to therapy were observed. It is important to note that patients in the study were already heavily pretreated and many underwent chemotherapy, radiotherapy, and surgical regimens without therapeutic effect. Moreover, peripheral blood mononuclear cells (PBMCs) from patients enrolled in the second phase of this trial, who were treated with IL-12, or patients with progression-free survival over 100 days showed an increase in ADCC against tumor cells coated with Cetuximab. It is possible, as suggested by the authors in this study, that Cetuximab may exhibit an off target effect by increasing the frequency of CTLA-4^+^ Tregs in the TME, thereby suppressing NK cell function (194). Accordingly, treatments incorporating anti-CTLA-4 mAbs or transient Treg depletion could potentially increase efficacy (195). Cetuximab in conjunction with toll-like receptor agonists, PD-1 blockade, and CD137 agonists, may also enhance efficacy, in addition to cytokine administration (69, 181, 182, 196–200). Moreover, combination treatment with super agonist cytokines (discussed below) may substantially potentiate Cetuximab activity, as recently described for the IL-15 super agonist ALT-803 (201). Interestingly, treatment with Nimotuzumab, a different anti-EGFR mAb, also promoted cross-talk between NK cells and DCs, leading to EGFR-specific CD8^+^ T-cell priming, and unlike Cetuximab, did not increase the abundance of Tregs (202). Nevertheless, both Cetuximab and Nimotuzumab may upregulate the expression of TIM-3 on the NK cell surface (202), potentially leading to exhaustion. Thus, the anti-EGFR pathway may require additional investigation in future treatments and could possibly be bypassed through a combination treatment of Cetuximab/Nimotuzumab with anti-TIM-3 antibodies.

*Anti-HER2: Trastuzumab*. Trastuzumab is a clinically used anti-HER2 mAb commonly used for treating HER2^+^ tumors. NK cells can mediate ADCC against Trastuzumab coated target cells and recruit T-cells through chemokine secretion. Trastuzumab treatment is also associated with high numbers of tumor infiltrating NK cells (203–205), and higher NK cell function and expansion may also correlate with response to Trastuzumab treatment (206, 207). A recent phase I clinical study used adoptive NK cell therapy together with Trastuzumab administration, showing good tolerability, and induction of immune activity (208). Nevertheless, several patients still display intrinsic and acquired resistance to Trastuzumab therapy (209). Interestingly, a recent study demonstrated that the number of circulating CD57^+^ NK cells from primary breast cancer patients receiving Trastuzumab therapy correlates with resistance to therapy (210). These NK cells showed reduced CXCR3 expression and proliferation, suggesting a phenotype exhibiting poor homing to and survival in the tumor niche. CD57^+^ circulating NK cells may therefore provide a novel prognostic biomarker for Trastuzumab treatment, and it would be interesting to consider reinvigorating this NK subset to increase the efficacy of Trastuzumab administration; it is possible that approaches involving upregulation of chemokine receptors (211) (such as CXCR3) or utilization of antibody-based homing proteins designed to increase NK cell homing along chemokine gradients (212) may restore their redirection to tumors, however a broader understanding of the molecular mechanisms and surface receptors governing the responsivity of the subset during Trastuzumab treatment are required. Enhancing the activation and proliferation of NK cells may thus benefit anti-HER2 targeting approaches. Indeed, aside from cytokine administration (IL-2/15/12) and glyco-optimization, additional approaches, which include anti-PD-1/TIGIT blockade, and CD137/Toll-like receptor agonists have been shown to potentially augment Trastuzumab efficacy in combinatorial studies (213). Another effective strategy to improve NK cell ADCC is incorporation of Trastuzumab in the bispecific antibody format (discussed below). Bispecific Trastuzumab antibody [(HER2)2xCD16] significantly enhanced ADCC compared to Trastuzumab treatment (214).

*Anti CD20: Rituximab/Obinutuzumab*. Rituximab and Obinutuzumab are clinically utilized mAbs targeting CD-20, which is expressed on the surface of mature B-cells and on the surface of most malignant B-cells (215). Evidence for the involvement of ADCC in the mechanism of Rituximab stems from the correlation between FcγR polymorphism and clinical responses in some hematologic malignancies, in addition to lack of therapeutic effects in FcγR deficient mice (216–218). Therefore, in order to improve effector function mediated by mAbs targeting CD-20, Obinutuzumab was glyco-modified to reduce Fc fucosylation (219). Indeed, Obinutuzumab demonstrated superior ADCC in xenograft models and *in-vitro* efficacy assays compared to Rituximab (218). Furthermore, the glyco-engineered Obinutuzumab appears to be less affected by the inhibitory effects of KIR/HLA ligation compared to Rituximab (220). Several lines of evidence, from *in-vitro* and animal studies show that NK cells are involved in the mechanism of action of Rituximab/ Obinutuzumab (221–224), yet their exact role in clinical settings are not completely understood. A recent clinical study suggested that lower peripheral NK cell counts in follicular lymphoma and lower peripheral and tumor infiltrated NK cell numbers in diffuse large B-cell lymphoma associate with transient progression-free survival in response to therapy (225). NK cell count could therefore potentially serve as a prognostic biomarker for efficacy of anti-CD20 treatment. As additional means to augment NK-mediated anti-CD20 therapy (apart from glyco-optimization), combination with cytokines such as IL-15/21 was shown to increase the efficacy of anti-CD20 treatment (226–228). Lenalidomide, an immunomodulatory drug commonly used for multiple myeloma also enhances the effect of anti-CD20 administration on NK cell effector function, in part by promoting nanoscale reorganization of the cytoskeleton at the immunological synapse (229–231). Moreover, a recent study revealed a CD56^hi^ CD16^−^ PD-1^hi^ NK population in HL patients. It is tempting therefore to speculate that anti-CD20 treatment might synergize well with PD-1/PD-L1 blockade in HL (88), and that incorporation of modulatory drugs such as lenalidomide may even enhance these combinations further.

##### mAbs in clinical trials- targeting tumor ligands for optimization of NK cell activity

Though it is clear that NK cells mediate some of the effects of clinically utilized immunotherapeutic mAbs, additional clinical studies are required to understand the full extent of their contribution. In addition to the caveats of mAb therapy mentioned above, it has become clear that reduction of ligand expression (internalization and shedding) and ligand specificity are critical parameters, which are starting to be addressed in recent pre-clinical and clinical studies (232).

One of the major challenges of current treatments for acute lymphoblastic leukemia (ALL), which target CD19 (such as CAR T-cell therapy), is the loss of CD19 surface expression (233). Therefore, identification of additional antigens that retain their expression on drug-resistant and relapsed ALL cells, may provide additional avenues for treatment (234). A recent study addressed this need through an optimized anti- B-cell activating factor receptor, BAFF-R antibody (VAY736), based on BAFF-R overexpression on B-ALL cells (234). Drug resistant and relapsed ALL cells maintained expression of BAFF-R, and VAY736 treatment decreased disease burden and increased NK cell activity. Nevertheless, advanced stages of the disease showed resistance to therapy, possibly due in part to NK cell exhaustion. Thus, patients with advanced stages of ALL may benefit from additional combinatorial treatments. Indeed, resistance to VAY736 therapy could be overcome with combination treatment using anti TGF-β antibody in this study. VAY736 is currently in phase I trials for CLL (NCT03400176), and it will be interesting to determine if it may synergize well with other ICIs that could restore NK cell functionality in patients.

In acute myeloid leukemia (AML), Krupka et al. demonstrated high CD157 expression on a majority of primary AML cells in samples from both newly diagnosed and relapsed patients. Since the antigen is not rapidly internalized, it provides a stable and attractive target for potential antibody-based therapy for AML. Utilizing a non-fucosylated anti-CD157 antibody (MEN1112) can induce potent anti-leukemic activity mediated through NK cells *ex-vivo* (235). Though MEN1112 showed promising effects mediated through NK cells against AML cell lines and primary AML cells, it had varying effects on NK cells derived from AML patients, emphasizing that NK cells from AML patients are functionally impaired. A recently described subset of ILC1-like cells in AML patients, with NK cell-like cytotoxic capabilities is functionally impaired by high levels of TGF-β in patients' serum and high HLA-E expression on leukemic blasts (236). Therefore, MEN1112 treatment may benefit from specific timing of administration during disease progression, and may also benefit from additional combinatorial approaches such as ICI (NKG2A blockade)/TGFβ blockade to negate NK cell exhaustion and to maximize efficiency. A phase I study for relapsed/refractory AML patients using MEN1112 (NCT02353143) is expected to shed light on its tolerability and potential future use in single/combination therapy.

NK cells are largely excluded from the TME in HL, and peripheral blood and tumor infiltrating NK cells from HL patients are severely impaired (237). Therefore, enhancing their activity in HL may greatly benefit treatment. A recent study describes targeting CD123 (IL-3Rα), expressed on most HL cells with an anti-CD123 antibody (CSL362) (238) to induce NK cell ADCC as a potential therapy (239). CSL362 incorporates the S239D and I332E substitution in the Fc region to improve binding affinity (240). This study demonstrated for the first time the feasibility of targeting HL through NK cells using antibody therapy. CSL362 increased primary NK cell mediated-killing of HL targets, though it stimulated high-affinity Fcγ-receptor NK-92 cells (haNK cells) to a greater degree than primary NK cells isolated from donors (239). Ernst et al. suggest that as haNK cells are less impacted by CD16 polymorphism, it is possible that they display higher cytotoxicity compared to primary NK cells. Thus, it currently appears that this treatment may be most effective in combination with adoptive NK cell treatment. It would be informative to analyze Fc polymorphism of the cohorts in a clinical setting to determine if indeed this is the major mechanism explaining discrepancy between autologous NK and haNK cells. Furthermore, analysis of NK cell subsets from HL patients may reveal additional avenues of treatment that may synergize with CSL632. CSL362 is being evaluated in a phase II clinical study for patients with myelodysplastic syndromes (NCT03011034).

##### mAbs in pre-clinical development–targeting tumor antigens for augmentation of NK cell activities

Previous clinical trials for AML showed limited efficacy when antigens such as such as CD123 (241) were targeted for treatment. Moreover, CD33 was also targeted with mAb therapy for AML, yet its limited clinical efficacy may have been caused by antigenic shift (242). Therefore, antibody engineering and targeting of different antigens may enhance NK cell activity and efficacy of treatment. Koerner et al. previously reported an optimized anti-CD133 antibody, 293C3-SDIE for AML, which was also recently tested by Schmied et al. for colorectal cancer (176, 243). 293C3-SDIE was engineered with Fc portion containing the S239D and I332E substitutions to enhance binding affinity for ADCC. In the AML study, 293C3-SDIE significantly decreased AML burden in xenograft mouse models, and was superior in anti-leukemic activity *in vitro* compared to WT 293C3. Interestingly, there appears to be large variability in the anti-leukemic effects of 293C3-SDIE, possibly due to differing expression patterns of CD133 between patients. This may also be in part due to evidence of HLA dimorphism and NK cell ligand expression impacting AML therapy (244, 245). Furthermore, as described above, autologous patient NK cells may have been functionally impaired, relative to allogeneic NK cells (176). Additional examination of NK cell receptor repertoire from healthy individuals and patients may be informative, to possibly screen for specific checkpoints that could synergize with 293C3-SDIE. Though the colorectal study utilizing 293C3-SDIE was conducted *in-vitro*, the preclinical data demonstrate it has a potent effect on NK cell activity against colorectal cell lines (243). Though additional study is required to test the effect of 293C3-SDIE against physiological solid tumors, this treatment may have important significance for NK cells against the CD133 tumor antigen, which is implicated in multiple cancer types (246). It would be interesting to follow effects of this antibody in future clinical settings, to validate efficacy and the lack of off-target toxicity against healthy cells expressing CD133, such as healthy hematopoietic progenitor cells and others (246, 247).

An anti-IL-7 receptor (IL-7Rα) antibody, B12, has also recently been described to induce potent NK cell-mediated ADCC against T-ALL (177). Studies previously showed that IL-7 is an important factor in the progression of T-ALL, enhancing cell expansion and survival (248), making it an attractive target for antibody therapy. In addition to stimulating NK cell ADCC, B12 appears to be rapidly internalized and trafficked into lysosomes of leukemic cells, demonstrating its additional therapeutic potential, such as for intracellular drug delivery. B12 administration into mice was not sufficient to halt the progression of the disease but did prolong mouse survival. Thus, in addition to NK cell stimulation, the treatment could be beneficial against disease in combination with additional drugs such as dexamethasone, which the authors reported to have an additive effect with B12 (177). It is also possible that blocking pathways that were reported to interfere with T-ALL progression using small molecule inhibitors, such as ones targeting JAK/STAT signaling pathways, may also synergize well with anti IL-7Rα mAb treatments (249). Moreover, a recent report showed that NK cells from T-ALL patients have reduced expression of activating receptors and reduced terminal differentiation, which may be bypassed by pre-activation of autologous NK cells with IL-12, IL-15, and IL-18 (250). Therefore, combining B12 with pre-treated NK cells may potentially show synergistic effects.

An approach that may synergize well with additional antibody-based treatments aims at maintaining tumor antigens on the cancer cell surface in order to abrogate their shedding or internalization. A recent study aimed at increasing NK cell activity against cancer cells utilized antibodies against the proteolytic shedding domains of MICA and MICB (the tumor ligands for NKG2D), which are often shed by the TME to inhibit NK cell function (178). This strategy was shown to prevent MICA/B shedding and to increase their expression on the tumor surfaces, ultimately inducing potent NK cell activity and inhibiting tumor growth (178). This approach may be applicable to several cancer types that upregulate MICA/B antigens and may have an additive effect with additional treatments. It is interesting to suggest that additional ligands [e.g., PVR and NKp30 ligands (232)] that are shed by cancer cells may also be targeted in a similar fashion to increase ADCC. Moreover, studies focused on CD16-mediated-killing demonstrate that CD16 undergoes significant proteolytic downregulation following activation (251). CD16 shedding is regulated through metalloproteases such as ADAM17 (252, 253). This mechanism may serve to facilitate NK cell detachment from targets and thereby increase their mobility (254). Inhibition of CD16 shedding through ADAM17 blockade may increase NK cell activation in the TME, thereby potentiating antibody treatments targeting NK cells (251, 255, 256). Sustaining this CD16 mediated activity may be especially important, as the Cerwenka group showed that NK cell engagement through CD16 might prime NK cells against cancers by providing them with memory-like effects (257).

Additional antibodies potentially involving NK cell activation and ADCC as mechanism of action were reviewed by Wang et al. (175). More recent reports of NK cell activating antibodies with varying combinatorial treatments are listed in Table 2, including anti-SLAMF7, Elotuzumab, for multiple myeloma (258); anti-cathepsin-D, F1, for Triple Negative Breast Cancer (179); anti-GD2, Dinutuximab and hu14.18K322A, for neuroblastoma (259, 260); anti-PD-L1, Avelumab, for Triple Negative Breast Cancer (261); anti-CD38, Daratumumab, for multiple myeloma (262, 263); anti- GPC3, Codrituzumab, for hepatocellular carcinoma (264); and anti-CD33, BI 836858, for AML (265).

#### Engagers of NK Cells to Unleash NK Cell Activity

BiKEs and TriKEs are multi-specific antibodies composed of a single-chain variable fragment from the heavy and light variable chains of an antibody that are joined by a short peptide linker and connected to the single-chain variable fragment of an additional antibody (BiKE) or two antibodies (TriKE) of interest. Usually, one of the components incorporates an anti-CD16 moiety to induce NK cell-mediated ADCC (266, 267). Thus, BiKEs and TriKEs can overcome the limitations described above when utilizing standard antibodies to induce ADCC. For example, the affinity of CD16 for the IgG Fc region of therapeutic antibodies of different isotypes can be increased by incorporating anti-CD16 into BiKEs and TriKEs (268). Additional advantages include superior bio-distribution, low immunogenicity, and relatively rapid construction (266).

Utilization of BiKEs and TriKEs to boost NK cell activity as a therapeutic approach was demonstrated in multiple malignancies, among which are Non-HLs, leukemia, metastatic breast cancers, EGFR expressing tumors, and carcinomas (266). The Vivier group recently showed the potency of a multifunctional NK cell engager composed of two antibody domains targeting, on the one hand, the activating NK cell receptor, NKp46, and on the other hand, specific antigens such as CD19, CD20, and EGFR (172), with an additional optimized Fc fragment for the binding of CD16. The multifunctional NK cell engager was injected into mice at different concentrations after tumor inoculation, and demonstrated enhanced NK cell infiltration into tumors and promoted tumor clearance in *in-vivo* models. Importantly, this study demonstrated how NK cell engagers could surpass the efficacy of current antibodies in clinical use, such as Rituximab, Obinutuzumab, and Cetuximab in *in-vitro* and *in-vivo* models. An additional important aspect of this technology is that in addition to promoting NK cell infiltration, it leverages the expression of NKp46 in the tumor microenvironment of multiple solid tumors, overcoming the issue of low CD16 expression on tumor-infiltrating lymphocytes (269). Moreover, harnessing the activating potential of multiple stimulatory receptors (CD16 and NKp46) on the NK cell surface can synergize to overcome inhibition, and fully potentiate NK cell activity (270). Thus, it would be interesting to follow this technology in clinical settings, and observe how different potential modifications such as alternative targeting moieties may be optimal for treatment of certain solid/hematological tumors.

One potential modification for improving efficacy of NK cell engagers is the inclusion of IL-15 moieties to induce NK cell proliferation and activation. TriKE 161519 was recently described by Felices et al. (271) for treatment of chronic lymphocytic leukemia (CLL); this antibody consists of anti-CD19 and anti-CD16 fragments, with an additional IL-15 moiety. Inclusion of the IL-15 moiety may be beneficial in CLL patients, who display low numbers and survival of mature and functional NK cells (272). TriKE 161519 induced superior NK cell activity against lymphoma cell lines and primary CLL patient cells compared to Rituximab, and, importantly, promoted NK cell survival and proliferation. The IL-15 moiety on this TriKE may also reduce off-target toxicities caused by general IL-15 administration by activating NK cells selectively through CD16. Similarly, previously described TriKE constructs (TriKE 161533, and TriKE 1615133) (273), which incorporate an IL-15 linker and contain anti-CD16 and anti-CD33 fragments (161533 TriKE), or anti-CD16 and anti CD133 fragments (1615133 TriKE), were described for activating NK cells against neoplastic mast cells, myelodysplastic syndrome cells, and cancer stem cells (273–275), as well as providing NK cells with sustained survival and proliferation signals. The plasticity of NK cell engagers enables them to simultaneously target additional moieties, as demonstrated by Schmohl et al., who constructed a TetraKE that binds CD16 on NK cells, EpCAM on carcinoma cells, and CD133 on cancer stem cells, while containing an IL-15 linker (1615EpCAM133) (276). This construct improved NK cell-mediated ADCC, NK cell survival, and NK cell proliferation without excessive IFN-γ secretion. Furthermore, the simultaneous targeting of the small cancer stem cell niche (with the anti-CD133 moiety), which is resistant to most conventional therapies, could potentially be a powerful addition to other mAb-based approaches. Current NK cell engagers appear to enhance NK cell therapy in a safe manner by targeting tumor antigens, sustaining NK cell proliferation and survival, surpassing some clinically utilized mAbs. Clinical trials testing TriKE 161533 will be starting imminently (NCT03214666), and it will be interesting to observe the development and clinical progress of the additional constructs.

The challenges facing therapy involving NK cell engagers include the complexity of the design process, screening, production yield, and selection of the proper tumor antigen (277). Often, tumor antigens are expressed on otherwise healthy tissue, adding an obstacle to possible on-target/off-tumor toxicity, requiring proper screening prior to development (17). Variance in CD16 affinity for Fc IgG, CD16 polymorphism, and varying expression of CD16 on tumor-infiltrating lymphocytes also remain a challenge, perhaps to be overcome by Fc glyco-optimization and targeting alternative activating receptors on the NK cell surface, such as NKp46. Furthermore, the proteolytic cleavage of CD16 during NK cell activation is also an issue that may be overcome by using metalloprotease inhibitors in combination with current treatments (253, 267).

### Sensitizing Tumors for NK Cell Killing

Additional strategies may synergize with current mAb therapy/ICI therapy by reducing the NK cell activation threshold. This could be achieved by masking tumor checkpoint ligands [i.e., anti-PD-L1, Avelumab, which can also initiate NK cell ADCC (69, 120, 169, 261, 278, 279), or the TIGIT ligands CD112/CD155 (105, 280, 281)], activating NK cells *in-situ* with cytokine variants, and modulating tumors via bio-chemical modifications or inhibitors, making them more susceptible to NK cell-mediated lysis.

#### Cytokines for NK Cell Activation in the TME

As mentioned above, systemic administration of cytokines such as IL-2/IL-12/ IL-15 is associated with adverse toxicities. Moreover, though IL-2 stimulates NK cell proliferation and activation, it concurrently enhances Treg expansion through the high affinity IL-2 receptor subunit-α (IL-2Rα) expressed on Tregs; these cells downregulate NK cell activity through secretion of TGF-β (76). In order to circumvent Treg expansion, much effort has been directed at generation of cytokine variants. Prominent examples includes the IL-2 “superkine,” with increases binding for IL-2Rβ expressed on resting T effector and NK cells, which can preferentially activate them without Treg expansion, and PEGylated IL-2, which preferentially activates IL-2Rβ (282, 283). Furthermore, IL-15 can substantially increase NK cell expansion and activation without stimulating Treg development; however it exhibits a short half-life *in-vivo* and thus requires frequent dosing for efficacy (284). Therefore, an IL-15 super agonist (ALT-803) was generated with a longer *in-vivo* half-life, and it is being tested in 25 clinical trials at the recruiting and active stages (www.clinicaltrials.gov) (285–287). Nevertheless, delivery of immune-stimulatory cytokines directly to the TME for local enhancement of NK cell survival and proliferation may be preferable to avoid systemic adverse immune events and to promote immune cell activation *in-situ*. Hutmacher et al. recently reported utilization of the fusion protein F8-IL2, consisting of IL-2 fused to the F8 antibody, specific to the alternatively-spliced EDA domain of fibronectin, which is expressed in multiple solid tumors and lymphomas (288), to increase NK cell activity in murine models. F8 can therefore direct IL-2 activation of NK cells to the TME. F8-IL2 was most effective when used in combination with immune checkpoint blockade (anti-CTLA-4) to increase NK cell infiltration and to unleash NK cells in the TME, delaying tumor growth in mouse models. Besides minimizing potential autoimmune toxicities, this approach can also increase the time window of cytokine stimulation overcoming the relatively short half-life of IL-2 (289). Conversely, inhibitory immunomodulatory cytokines such as TGF-β may be targeted *in situ*, specifically in the TME, to reduce their suppressive effects on NK cells. Once again, specific targeting of TGF-β in the TME is important due to potential toxicities associated with anti-TGF-β therapies administered systemically (290). Knudson et al. reported an anti-PD-L1/TGF-β fusion protein (M7824), which depletes TGF-β signaling in the TME. This treatment promoted an activated NK cell phenotype and tumor clearance in murine models (291). Thus, this treatment appears to have a dual effect, both blocking PD-L1 and sequestering TGF-β to overcome tumor resistance to immune cell function. In initial clinical trials, M7824 appeared to be well-tolerated in patients with pre-treated advanced solid tumors (NCT02517398) with early signs of efficacy (292), and is involved in multiple clinical trials as part of different combination therapies (www.clinicaltrials.gov).

#### Novel NK Cell Inhibitory Checkpoints/Tumor Antigens for Blockade

Novel NK cell checkpoint ligands expressed on cancer cells could be desirable targets for new immune checkpoint blockers with favorable toxicity profiles compared to other established immune checkpoints (171). Recently, it was demonstrated that the NKp44 isoform1 receptor, a splice variant of the activating NKp44 receptor, can bind membrane proliferating cell nuclear antigen (PCNA) upregulated on cancer cells, inhibiting NK cell activation (293, 294). This is a potentially attractive target due to its different expression profile in healthy proliferating cells vs. cancer cells. In healthy cells, PCNA appears to be expressed in the nucleus and is involved in DNA replication and repair (295), whereas in cancer cells, it is overexpressed on the cell surface (296). By constructing a monoclonal antibody against PCNA (14-25-9), the Porgador group was able to demonstrate that blocking this tumor antigen unleashes NK cell activity *in-vitro* and *in-vivo*, and promotes tumor clearance in murine xenograft models of autologous HNSCC patient NK cells (294). It is interesting to suggest that splice variants of additional activating NK receptors may restrain NK cell activity when interacting with atypical ligands, and their expression on tissue resident/tumor infiltrating NK cell subsets should be characterized. This could potentially provide multiple new avenues for mAb-mediated blockade and combinations with established mAb therapy. Thura et al. have also recently taken advantage of high cell surface expression of the oncogenic phosphatase of regenerating liver 3 (PRL3) on hepatocellular carcinoma cells for targeted therapy. Overexpression of PRL3 is associated with heightened tumorigenicity and metastatic spread and is thus an attractive immunotherapeutic target (297). Blocking PRL3 with a humanized antibody (PRL3-zumab) enriched NK cells in the tumor niche and promoted tumor clearance in mouse models (298). Since this study also demonstrated elevated expression of PRL3 on multiple patient-derived tumor samples with little expression on the matched healthy tissue, this therapeutic approach may be beneficial for additional malignancies, and may provide enhanced specificity for immunotherapy. PRL3-zumab is involved in two clinical trials for advanced solid tumors (NCT03191682, NCT04118114).

#### Turning “Cold” Tumors “Hot” for NK Cell Immune Surveillance With Synthetic Targeting

Identification of tumor-specific antigens for immunotherapy remains a difficult task due to the heterogeneity of the tumor and tumor infiltrating immunological niche, antigen internalization and shedding, antigen mutations, and differential expression profiles between patients (232, 299). Furthermore, many antigens on tumors may also be expressed on healthy tissue. Therefore, several recent studies suggested modulating tumors through synthetic biochemical modifications in order to enhance NK cell infiltration and function. Two recent studies demonstrate that tumors can be coated with antibodies and antibody Fc fragments, exposing them to NK cell-mediated ADCC. These studies take advantage of specific biochemical characteristics of the TME, such as lower pH levels and high sialic acid expression, to directly coat tumors with antibody fragments (300, 301). Subsequently, NK cells can recognize these Fc IgG fragments and become activated *in-situ* to promote tumor clearance in *in-vivo* models. The antibody coating treatments also appear to be non-toxic and to additionally promote adaptive immune responses against tumors. It would be interesting to consider whether additional ligands for NK cell receptors [such as NCRs to deliver synergistic signals (302)] can be coated together with Fc IgG fragments to provide additive effects and to ensure the safety and efficacy of these approaches in pre-clinical and clinical studies. Furthermore, combinations of treatments to stimulate NK cell expansion and activation (such as IL-15 administration) and checkpoint blockade may potentially enhance the efficacy of these approaches.

A similar recent approach includes induced expression of certain genes in tumors that may synergize with other immunotherapies. Meraz et al. demonstrated “forced” expression in tumors of tumor suppressor candidate 2 (TUSC2/ FUS1) by an engineered expression plasmid delivery system, in a mouse model of lung cancer. TUSC2 is reduced in a significant number of lung cancers, and this reduced expression is associated with worse prognosis (303). Therefore, counteracting this down-regulated state may be an effective strategy to sensitize tumors for immunotherapy. Indeed, forced induction of TUSC2 expression in lung cancer cells increased NK cell infiltration and stimulated NK cell activation. Interestingly, TUSC2 modulated the TME to facilitate homing and altered the infiltrating immune populations through an increase of IL-15 and of the chemo-attractants Ccl3/4. This is especially important due to low NK cell infiltration and proliferation in solid tumors, and may open avenues for targeting tumor suppressor genes to increase IL-15 in the tumor bed, since its expression by the pro-inflammatory milieu in tumors was recently shown to play a critical role in NK cell infiltration and activation (304). TUSC2 treatment promoted the survival of mice in the lung metastasis model, and synergized with conventional anti-PD1/CTLA-4 therapy (305). An active trial is currently underway with TUSC2 in combination with the EGFR inhibitor Erlotinib for stage IV lung cancer (NCT01455389). It would be interesting to evaluate TUSC2 administration in future clinical settings in combination with immune checkpoint blockade.

#### Upregulation of NK Cell Ligands

Additional therapeutic approaches harness chemical compounds that induce the expression of NK cell-activating ligands on tumors, or that inhibit tumor pathways promoting NK cell immunosuppression (306). Such strategies may increase tumor susceptibly to immune surveillance, and synergize with current therapies such as mAb treatment and checkpoint blockade, since downregulation of activating ligands promotes tumor escape (29). For example, recently reported approaches include upregulation of the activating NKG2D NK cell ligands on colon cancer cells, multiple myeloma cells, and hepatocellular carcinoma cells, through administration of spironolactone (SPIR) (307), upregulation of Liver X receptor (308), and inhibition of Enhancer of zeste homolog 2 (EZH2) (309). Previous studies also reported sensitizing tumors to NK cell activity by targeting alternate signaling pathways in tumors, including inhibition of BRAF (V600E) (310), STAT3 inhibition (311), and modulation of cancer cell autophagy (312, 313).

An approach that may activate NK cell activity in the TME and is being investigated in the clinic is inhibition of histone deacetylases (HDACs) (314). Through epigenetic alteration of their genomes, cancer cells may acquire advantages that facilitate their immune escape. HDAC inhibition may increase antigen presentation on the surface of cancer cells, making them more susceptible to immune cell surveillance, thereby potentially enhancing the efficacy of additional immunotherapies (315). Hicks et al. recently studied the use of HDAC inhibitors in conjunction with standard anti PD-1/PD-L1 therapy (316). HDAC inhibitors induced upregulation of the NKG2D stress ligands MICA/B on different carcinoma cell lines, increased the activated phenotype of patient-derived NK cells, and sensitized tumors to NK cell killing. Likewise, Stone et al. demonstrated use of DNA methyltransferase and histone deacetylase inhibitors on ovarian cancer cells, showing that this treatment increases NK cell infiltration and activation in the TME, as well as extending the life of animal models (317). Therefore, screening and identification of tumors with low NK activating ligand expression (such as MICA/B) in patients may favor utilization of HDAC inhibitors to increase their susceptibility to NK cell immune surveillance. Ongoing clinical trials involving HDAC inhibitors with anti-PD-1/PD-L1 therapy (NCT02708680, NCT02915523, and NCT02900560) may shed additional light on the efficacy and safety of these treatments.

Friedman et al. recently utilized an inhibitor for the WEE1 kinase (AZD1775) on head and neck cancer cells, which reversed G2/M cell cycle checkpoint activation of the cancer cells, resulting in increased DNA damage and susceptibility to NK cell-mediated killing (318). Interestingly, DNA cell cycle checkpoint in this study did not impact surface expression of MHC-I, PD-L1, or NKG2D ligands (RAE, H60, MULT-1) on cancer cells, despite increasing their susceptibility to NK cells. These data may suggest a global sensitization mediated through AZD1775 that could bypass the need for anti-PD-1 or anti-NKG2D checkpoint blockade, and it is interesting to consider utilizing it universally for multiple NK-based approaches on tumors with low PD-L1/NKG2D ligand expression. Stimulation of NK cells in the TME via administration of the Heparan sulfate (HS) mimetic Pixatimod (PG545) has also recently been reported. This treatment inhibits the tumor Heparanase enzyme, which promotes extracellular matrix degradation and tumor invasion/metastases. By activating DC production of IL-12, PG545 enhanced the anti-tumor function of NK cells in murine lymphoma models. Therefore, this treatment may synergize well with other immunotherapeutic treatments to stimulate NK cell activity in the TME due to the pleotropic effects of IL-12, and it is interesting to consider its utilization together with mAb therapies to maintain infiltrating NK cell activity. Pixatimod can also be utilized in combination with anti-PD-1 treatment to elicit an even greater anti-tumor effect (319, 320). Furthermore, inhibition of another common enzyme in the TME, arginase 1 (Arg1), using the small molecule inhibitor CB-1158 (clinical trials in combination with checkpoint inhibitors for advanced solid tumors- NCT02903914), was also shown to activate NK cells, promote their infiltration into the TME, and synergize with checkpoint inhibition to promote tumor clearance. This pathway is mediated through inhibition of MDSCs in the TME, which express Arg1 and lead to a decrease of L-arginine, which is required for NK cell proliferation and function (321). It is possible that enzymes such as Arg1 and IDO-1 (322) confer some resistance of tumors to current treatments such as checkpoint blockade, by depleting essential metabolites such as arginine and tryptophan in the TME. Therefore, identification and targeting of novel inhibitory pathways in the TME is warranted, and may significantly bolster the efficacy of current treatments if administered in combination. It is also tempting to consider simultaneous targeting of multiple suppressive pathways in addition to clinically utilized treatments, though first testing for efficacy and specificity of treatment.

#### Modulating Tumor Metabolites

Finally, modulation of metabolites in the TME appears to be an attractive strategy to increase anti-tumor NK cell activity. Cancer cells metabolize glucose more rapidly compared to normal cells, producing large amounts of lactate, in a process known as the Warburg effect (323). This metabolic shift has important ramifications on the TME, and thus, on tumor-infiltrating immune cells (324). One metabolite that is present at higher concentrations in the TME is adenosine. Studies show the inhibitory effect of adenosine accumulation in the TME on immune cell function in general, and NK cell activity, primarily through engagement with the adenosine receptor A_2A_R (325, 326). Adenosine signaling in the TME can reduce NK cell proliferation and activation, as well as its effector function (327–329). Blocking adenosine-catalyzing enzymes and purinergic signaling in the TME (such as antibodies against CD73, CD39, and CD38, which mediate the production of extracellular adenosine, and blockade of A_2A_R) is therefore being investigated in multiple clinical trials in combination with immune checkpoint blockade (330) and may synergistically enhance tumor-resident NK cell activity (330–338).

## Perspective

It is evident that NK cells elicit potent anti-tumor responses by complimenting T-cell recognition, and by initiating cross-talk with the tumor-resident immune-cell milieu. As recently demonstrated, they are an attractive addition to T-cell therapy due to their ability to trigger potent immune responses in the TME, promoting adaptive anti-tumor activity. Harnessing NK cells for immunotherapy is also highly attractive due to their innate ability to recognize transformed cells. Evidence is mounting demonstrating the potential for activation of NK cells *in-vivo*, and though we are beginning to understand the mechanisms that suppress NK cell immune surveillance in cancer, more research is required to clarify the circuits that ultimately dictate their capacity to elicit anti-tumor responses. Current *in-vivo* approaches mostly seek to increase the activation and expansion of the overall NK population. It would be interesting to consider, given our increasing understanding of NK cell subpopulations and NK cell tissue/intratumor residency, the harnessing of specific NK effector populations *in-vivo* that may generate the most robust anti-tumor responses, while avoiding tolerant subsets or regulatory ILCs, which can inadvertently hamper therapy. Furthermore, current approaches such as CAR-T therapy, ICI therapy, and small molecule inhibition are subject to adaptive and acquired resistance, resulting from the complex immune-cancer landscape and immune-editing (15). Alternatively, it may be possible that adopting a “bottom-up” approach, that is, first enhancing the innate immune landscape (such as NK cells and ILCs) over different time regimens, secondly, targeting the suppressive TME milieu to negate exhaustion, and finally, enhancing adaptive immune responses, could sustain greater anti-tumor effects. Though this is an intriguing concept, additional studies and safety measures must be implemented to evaluate its efficacy. It is likely that a combinatorial approach, involving more than one facet of therapy, will be most effective, and could hold great promise in clinical settings (339).

## Author Contributions

AB-S, GB, and MB-S wrote the manuscript.

### Conflict of Interest

The authors declare that the research was conducted in the absence of any commercial or financial relationships that could be construed as a potential conflict of interest.
